# Respiratory Bacteria and Antimicrobial Resistance Genes Detected by Long-Read Metagenomic Sequencing Following Feedlot Arrival, Subsequent Treatment Risk and Phenotypic Resistance in Feedlot Calves

**DOI:** 10.3390/antibiotics14111098

**Published:** 2025-11-01

**Authors:** Jennifer N. Abi Younes, Lianne McLeod, Stacey R. Lacoste, Zhijian Chai, Emily K. Herman, E. Luke McCarthy, John R. Campbell, Sheryl P. Gow, Paul Stothard, Matthew G. Links, Simon J. G. Otto, Cheryl L. Waldner

**Affiliations:** 1 Department of Large Animal Clinical Sciences, Western College of Veterinary Medicine, University of Saskatchewan, Saskatoon, SK S7N 5B4, Canada; 2 Department of Biochemistry and Medical Genetics, Max Rady College of Medicine, University of Manitoba, Winnipeg, MB R3E 0J9, Canada; 3 Department of Animal and Poultry Science, College of Agriculture and Bioresources, University of Saskatchewan, Saskatoon, SK S7N 5A8, Canada; 4 Canadian Integrated Program for Antimicrobial Resistance Surveillance, Public Health Agency of Canada, Saskatoon, SK S7N 5B4, Canada; 5 Department of Agricultural, Food, and Nutritional Science, Faculty of Agricultural, Life, and Environmental Sciences, University of Alberta, Edmonton, AB T6G 2P5, Canada; 6 HEAT-AMR (Human-Environment-Animal Transdisciplinary AMR) Research Group, School of Public Health, University of Alberta, Edmonton, AB T6G 2J7, Canada; 7 Centre for Healthy Communities, School of Public Health, University of Alberta, Edmonton, AB T6G 1C9, Canada

**Keywords:** bovine respiratory disease, bacteria, antimicrobial resistance, antimicrobial resistance genes, long-read metagenomic sequencing, feedlot calves

## Abstract

**Background/Objectives**: Long-read metagenomic sequencing can assign antimicrobial resistance genes (ARGs) to speciated bacterial reads. This study evaluated whether metagenomic data from respiratory bacteria derived from feedlot calves sampled in the early feeding period were associated with subsequent bovine respiratory disease (BRD) treatment and phenotypic antimicrobial resistance (AMR) at treatment. **Methods**: Deep nasopharyngeal swabs (DNPSs) obtained at arrival processing (1 day on feed; DOF), 13 DOF, and the time of BRD treatment were cultured and subjected to antimicrobial susceptibility testing (AST) and long-read metagenomic sequencing. Analyses focused on macrolide (*mphE-msrE*, *EstT*) and tetracycline (*tet(H*)) ARGs within reads assigned to *Mannheimia haemolytica*, *Pasteurella multocida*, *Histophilus somni*, or *Bibersteinia trehalosi*. Generalized estimating equations assessed associations between metagenomic results from 1 and 13 DOF and subsequent BRD treatment risk and AST outcomes at treatment, at both the individual animal (calf) and pen levels. **Results**: Calf-level detection of *H. somni* at 13 DOF was associated with a greater BRD treatment risk between 14 and 45 DOF. An increased pen prevalence of either *M. haemolytica* or *P. multocida* at 13 DOF was associated with a greater BRD treatment risk from 14 to 45 DOF. At 13 DOF, detections of *mphE-msrE*, *EstT*, or *tet(H)* in target bacteria were associated with corresponding phenotypic AMR at BRD treatment. Similarly, a higher pen-level prevalence of *mphE-msrE* or *EstT* at 13 DOF was also associated with increased macrolide resistance at BRD treatment. **Conclusions**: The results from long-read metagenomic sequencing of DNPSs collected at 13 DOF were associated with both BRD risk and AMR at treatment. These findings align with prior culture-based results and support the potential utility of pen-level metagenomic testing for AMR surveillance and informing antimicrobial selection in feedlots.

## 1. Introduction

The use of metagenomic sequencing for microbial detection and characterization is expanding, as it provides comprehensive insights into complex microbial communities. Metagenomic sequencing enables the simultaneous identification of diverse bacterial populations and antimicrobial resistance genes (ARGs), providing utility in both research and clinical settings. Thus, it is increasingly being used in the study of bovine respiratory disease (BRD) [1,2,3,4,5,6,7].

Bovine respiratory disease is the leading cause of morbidity and mortality in feedlot cattle, contributing to substantial economic losses and challenges in disease management [8,9]. BRD is a multifactorial disease that is influenced by bacterial, viral, environmental, management, and host factors. The most frequently documented bacterial pathogens are members of the *Pasteurellaceae* family, primarily *Mannheimia haemolytica*, *Pasteurella multocida*, and *Histophilus somni* [10,11,12], and potentially *Bibersteinia trehalosi* [13]. Given the limited evidence for the success of vaccines in calves at feedlot arrival [14], antimicrobial therapy remains the most effective intervention for BRD control and treatment [15,16,17,18]. Prudent antimicrobial stewardship in feedlots relies on making informed treatment decisions, ideally before clinical disease develops. While culture-based diagnostic methods remain the standard for informing antimicrobial use (AMU), they typically provide a delayed and incomplete profile of prospective AMR. Consequently, there is interest in exploring alternative diagnostic tools, such as sequencing-based technologies, to support earlier risk assessment and to guide prudent AMU [19,20,21].

While culture, antimicrobial susceptibility testing (AST), and metagenomic sequencing each have limitations, metagenomics offers the advantage of detecting both a broader range of pathogens and ARGs reflecting the potential for antimicrobial resistance (AMR) within the microbial community [22]. In addition, metagenomic technologies that produce longer read lengths enable the identification of target respiratory bacterial species and associated ARGs within the same raw read [22] to more directly support diagnostic interpretation, surveillance, and potentially treatment decisions. Phenotypic culture and AST results obtained at or near the time of feedlot arrival have previously been associated with AMR at the time of BRD diagnosis [23]. However, whether ARGs detected during the early feeding period can similarly predict phenotypic AMR status at the time of BRD treatment is unknown.

Feedlot cattle are group-housed and managed in pens, with the health of an individual being influenced by its pen mates [24,25,26,27,28]. Thus, pens provide an opportunity for microbial and ARG transmission between calves [29]. Previous work using culture and AST data found that the pen-level prevalences of specific bacteria with AMR patterns detected shortly after arrival were associated with a higher likelihood of the same resistance patterns observed in calves at the time of BRD treatment [23]. These findings underscore the importance of evaluating both individual- and group-level associations when using metagenomic sequencing to inform disease risk and treatment strategies.

The goal of this study was to evaluate whether targeted long-read metagenomic sequencing data from feedlot calves sampled at two time points in the early feeding period (1 DOF and 13 DOF) were associated with the risk of future BRD treatment, as well as phenotypic resistance (AST) at the time of the first BRD treatment. The first objective was to assess whether the detection of *M. haemolytica*, *P. multocida*, *H. somni,* or *B. trehalosi* at 1 DOF was associated with a higher likelihood of BRD treatment within the first 13 DOF, and then whether detection at 13 DOF was associated with BRD treatment between 14 DOF and 45 DOF. Our second objective was to evaluate whether, among calves that developed BRD, targeted ARGs within bacterial reads at 1 DOF and 13 DOF were associated with corresponding phenotypic resistance at the time of BRD treatment. The third objective assessed whether a higher pen-level prevalence of these sequencing targets at 1 DOF or 13 DOF was associated with a greater risk for a randomly selected calf from that pen developing BRD or having corresponding phenotypic resistance at treatment.

## 2. Results

### 2.1. Study Population and Descriptive Statistics

The phenotypic AST results for *M. haemolytica*, *P. multocida*, and *H. somni* at arrival processing (1 DOF), 13 DOF, and the time of the first BRD treatment from this cohort of feedlot cattle were previously reported [23,27]. For the 1599 cattle available for sampling over the two-year-long study, long-read metagenomic sequencing results were available for 840 calves at 1 DOF, 819 calves at 13 DOF, and 128 calves that received a first treatment for BRD. For the 840 calves with some sequencing data, the mean arrival weight was 238 kg (524 lbs) [range: 163–290 kg (358–638 lb); SD: 20 kg (44 lb)]. At arrival, calves that were later treated for BRD weighed on average 9.5 kg (21 lbs) less than calves that were never treated. However, when adjusting for pen-level clustering and year/metaphylaxis group, this difference was not statistically significant (*p* = 0.78).

Based on the first 12 digits of the individual animal radio frequency identification (RFID) tag numbers, calves with sequencing data were sourced from 347 unique farms of origin.

Of the 100 calves per pen originally available for sampling, metagenomic sequence data were generated for 426 calves at arrival in 2020 (pen 1, 63; 2, 56; 3, 52; 4, 51; 5, 52; 6, 50; 7, 52; 8, 50) and 414 (215 + 199) in 2021 (pen 9, 50; 10, 53; 11, 62; 12, 50; 13, 50; 14, 49; 15, 50; 16, 50). Similarly, at 13 DOF, metagenomic data were available in 2020 for 404 calves (pen 1, 50; 2, 51; 3, 51; 4, 50; 5, 51; 6, 50; 7, 51; 8, 50) and 415 in 2021 (pen 9, 50; 10, 53; 11, 62; 12, 51; 13, 49; 14, 50; 15, 50; 16, 50).

Among the 128 calves treated for BRD, the mean arrival weight (available for 127 calves) was 230 kg (506 lb) [range: 188–281 kg (415–618 lb); SD: 19 kg (41 lb)]. These calves were also from diverse origins; in 2020, 27 treated calves originated from 24 unique farms, while in 2021 the 101 treated calves were from 63 unique herds of origin.

Metagenomic sequencing data for *M. haemolytica*, *P. multocida*, *H. somni* (Supplementary Table S1), and associated ARGs (Table S2) were summarized in the companion paper [30]. Briefly, at arrival processing, ARG detection was rare across all organisms, with *tet(H)* being the most frequently observed gene. In 2020, *tet(H)* was identified in *M. haemolytica* reads in 12% of samples compared to ≤5% in *P. multocida* and *H. somni* reads. In 2021, ARG detection at arrival processing was ≤5% across all bacteria. By 13 DOF, ARG detection increased, but differed by year and metaphylaxis protocol. In 2020, 33% of samples had *M. haemolytica* reads with *msrE-mphE*, while this combination was rarely detected at 13 DOF in 2021 (0% samples from oxytetracycline-treated calves; 0.5% in samples from tulathromycin-treated calves). Additionally, in 2020, *tet(H)* was identified in *M. haemolytica* reads from 14–21% of samples, and *EstT* in 8%. *EstT* was also identified in *H. somni* from 15% of samples at 13 DOF in 2020, though ARG detection in *H. somni* was otherwise uncommon. In 2021, *EstT* was detected in *M. haemolytica* from 14% of samples from tulathromycin-treated calves and <4% of oxytetracyline-treated calves. *P. multocida* and *H. somni* reads otherwise carried ARGs less frequently, with *tet(H)* most often observed in oxytetracycline-treated calves (12% and 5%, respectively) and *EstT* limited to ≤7.5%. Overall, ARGs were generally most frequently identified on reads classified as *M. haemolytica* at each time point, while *P. multocida* and *H. somni* contributed smaller proportions.

Data for *B. trehalosi* and calves with BRD had not been previously reported, and as such Table S1 provides metagenomic sequence statistics that include these groups. Compared to the other bacteria, *B. trehalosi* was detected at a lower abundance, with median read counts of 26 in 2020 and 2 in 2021, and median total base pairs of 51,105 and 6234, respectively.

*B. trehalosi* was detected through metagenomic sequencing in 26% of DNP samples at arrival processing, 14% at 13 DOF, and 18% from calves treated for BRD, with detection generally less frequent than the other *Pasteurellaceae*. ARG detection was uncommon in *B. trehalosi* reads and varied by year and metaphylaxis group (Table S2). In 2020, the most common ARGs detected in *B. trehalosi* reads were *msrE-mphE*, identified in 18% of calves at 13 DOF, while *EstT* and *tet(H)* were rarely identified (<1% and 3.5%, respectively). In 2021, ARG detection in *B. trehalosi* reads was minimal, with <3% detection of *tet(H)* across both years and metaphylaxis groups and no *msrE-mphE* or *EstT* detected. By also considering *B. trehalosi* reads as part of the metagenomic data, *msrE-mphE* were detected in three samples that were negative for ARGs in the other target *Pasteurellacea*, and *tet(H)* was detected in seven additional samples.

At the time of BRD treatment, ARG detection was most frequent in *M. haemolytica* reads; particularly in 2020, where *msrE-mphE* was identified on *M. haemolytica* reads in 48% of samples, followed by *tet(H)* in 26% and *EstT* in 19% (Table S3). *H. somni* reads with *EstT* were also detected in 19% of samples. The detection of ARGs across the other bacteria in calves treated for BRD in 2020 was less common (<20%) (Table S3). In 2021, ARG detection in samples from BRD-treated calves differed by metaphylaxis group. In oxytetracycline-treated calves, *tet(H)* predominated most frequently in *M. haemolytica*, but was also present in *P. multocida*, *H. somni*, and *B. trehalosi* (Table S3). Because *B. trehalosi* reads were also evaluated, *tet(H)* was detected in two additional samples from sick calves that would have been missed if only the other *Pasteurellacea* were considered. In tulathromycin-treated calves, all three ARGs were detected in samples from calves treated for BRD with *M. haemolytica*, while only *tet(H)* was detected in *H. somni*, and no ARGs were detected in *B. trehalosi*.

### 2.2. Association Between Metagenomic Detection of Bacteria at Arrival Processing and 13 DOF and Calf-Level BRD Treatment

For the 839 calves with DNPS samples sequenced at arrival processing, the detection of *M. haemolytica*, *P. multocida*, *H. somni*, or *B. trehalosi* prior to metaphylaxis was not associated with an increased likelihood of BRD treatment within the first 13 DOF (Table 1a).

In contrast, among the 819 calves with sequencing data available at 13 DOF, the detection of *H. somni* was associated with approximately double the likelihood of receiving BRD treatment between 14 and 45 DOF (OR = 2.1, *p* = 0.04) (Table 1a). The detection of *M. haemolytica*, *P. multocida*, or *B. trehalosi* in individual calves at 13 DOF was not associated with a subsequent BRD treatment risk between 14 and 45 DOF. However, higher pen-level prevalences of *M. haemolytica* and *P. multocida* at 13 DOF were both associated with greater likelihoods of BRD treatment between 14 and 45 DOF (Table 1b).

### 2.3. Among Calves Treated for BRD ≤ 13 DOF, Association Between ARG Detection at Arrival Processing and Phenotypic Resistance at BRD Treatment

For the 64 calves diagnosed and treated for BRD at or before 13 DOF, no calves had *Pasteurellaceae* (*M. haemolytica*, *P. multocida*, *H. somni*, or *B. trehalosi*) with *mphE-msrE* detected within sequencing reads at arrival processing. One calf had *EstT* detected within a target bacterial read, and three calves had *tet(H)* detected.

As no calves treated for BRD had bacterial reads with *mphE-msrE* at arrival processing, the associations between the sequencing-based detection of these genes and macrolide resistance at the time of BRD treatment could not be evaluated under either MIC categorization approach (Table 2). Additionally, while the categorization of intermediate MICs affected the number of non-susceptible isolates, none of the examined ARGs at arrival processing were associated with phenotypic susceptibility results at the time of BRD treatment, regardless of MIC interpretation (Table 2).

### 2.4. Among Calves Treated for BRD 14–45 DOF, Association Between ARG Detection at 13 DOF and Phenotypic Resistance at BRD Treatment

Sixty-four calves were diagnosed and received a first treatment for BRD between 14 and 45 DOF (Table 3a). The associations between the detection of *mphE*-*msrE* within *Pasteurellaceae* reads and an increased likelihood of resistance to 15-membered ring macrolides (tulathromycin and gamithromycin) were consistent across both categorization approaches for intermediate MIC values (Table 3a). Very similar results were observed when evaluating the association between the pen-level prevalence of *mphE*-*msrE* at 13 DOF and the detection of resistance to 15-membered ring macrolides in calves that were treated for BRD between 14 and 45 DOF (Table 3b). Neither the detection of *mphE-msrE* at 13 DOF nor the pen-level prevalence of *mphE*-*msrE* at 13 DOF were associated with resistance to 16-membered macrolides at the time of subsequent BRD treatment (Table 3a,b).

When susceptible and intermediate MIC values were categorized together (Table 3ai), the sequencing detection of *EstT* was associated with an increased odds of resistance to any macrolide (OR = 6.3, *p* = 0.001), 15-membered macrolides (OR = 6.3, *p* = 0.001), and 16-membered ring macrolides (OR = 7.4, *p* = 0.02). When intermediate results were recategorized as resistant (Table 3aii), one additional calf with *EstT* detected in the bacteria of interest had a non-susceptible isolate, and eight calves without *EstT* detection had isolates that were reclassified from the susceptible with intermediate categorization to non-susceptible (intermediate with resistant). Accordingly, the odds ratios decreased, and the association with 16-membered macrolide resistance at the time of treatment was no longer significant (OR = 3.3, *p* = 0.28), while associations remained significant for any macrolide (OR = 14, *p* = 0.003) and 15-membered macrolides (OR = 3.6, *p* = 0.01).

The association between pen-level prevalence of *EstT* at 13 DOF and 16-membered ring macrolide resistance at the time of subsequent treatment was significant regardless of how intermediate resistance was classified (Table 3b). There were no associations between pen-level *EstT* prevalence at 13 DOF and 15-membered ring macrolide resistance in calves treated for BRD between 14 and 45 DOF (Table 3b).

The detection of *Pasteurellaceae* with *tet(H)* at 13 DOF was associated with subsequent phenotypic tetracycline resistance at the time of BRD treatment between 14 and 45 DOF, when susceptible and intermediate MIC values were categorized together (OR = 11, *p* = 0.004) (Table 3ai). However, when intermediate MIC results were categorized as resistant (Table 3aii), one additional calf with *tet(H)* detected in bacterial reads had an isolate categorized as non-susceptible, and six calves without *tet(H)* detection were reclassified from the susceptible to the non-susceptible category. With the categorization of intermediate as non-susceptible, the odds ratio decreased, and the association between *tet(H)* detection at 13 DOF and subsequent tetracycline resistance at the time of BRD treatment was no longer significant (OR = 1.3, *p* = 0.78) (Table 3aii). There were no significant associations between the pen-level prevalence of *tet(H)* at 13 DOF and the detection of tetracycline resistance in calves treated for BRD from 14 to 45 DOF (Table 3b).

## 3. Discussion

These analyses explored the value of long-read metagenomic sequencing data from the early feeding period for assessing potential disease risks and forecasting AMR patterns in calves that subsequently develop BRD. This study demonstrated that the long-read metagenomic sequencing of DNPSs collected within two weeks on feed can provide data to support antimicrobial treatment decisions for feedlot cattle at risk of developing BRD. The longitudinal design included sequential sampling to evaluate whether the metagenomic-based detection of BRD-associated bacteria and ARGs in healthy calves could predict a subsequent BRD diagnosis and phenotypic resistance. In addition to evaluating individual-level associations, the pen-level prevalence of these sequencing targets was also assessed to determine whether pen-level prevalence was associated with higher odds of BRD or phenotypic resistance in individual calves from those pens.

The detection of *mphE-msrE*, *EstT*, or *tet(H)* within *Pasteurellaceae* (*M. haemolytica*, *P. multocida*, *H. somni*, or *B. trehalosi*) from individual calves at 13 DOF was associated with corresponding phenotypic resistance at the time of BRD diagnosis later in the feeding period. Moreover, these patterns were also observed when analyses were extended to the pen level. Pens with a higher prevalence of *mphE-msrE* or *EstT* were associated with a greater likelihood of detecting 15- or 16-membered ring macrolide resistance, respectively, in individual calves from those pens that later required treatment. Prior work showed that phenotypic resistance in bacteria recovered from fall-placed calves sampled at 13 DOF was associated with a subsequent detection of the same bacterial resistance patterns at the time of BRD diagnosis [23]. The present study extends this work by demonstrating that ARG detection using long-read metagenomic sequencing in healthy calves can similarly predict the isolation of bacteria with corresponding phenotypic resistance at the time of BRD diagnosis and treatment.

The decision to focus on *mphE-msrE*, *EstT*, and *tet(H)* in this analysis was guided by the prior literature linking these ARGs to macrolide and tetracycline resistance in *Pasteurellaceae* [31,32,33,34], as well as these drugs being commonly used to manage BRD [16]. Specifically, *mphE* and *msrE* have been associated with a resistance to 15-membered ring macrolides such as gamithromycin and tulathromycin [35], while *EstT* has been described as being associated with resistance against 16-membered ring macrolides (tilmicosin and tildipirosin) [36] and *tet(H)* with tetracycline-class antimicrobials [37]. Accordingly, the current study explored whether the detection of these ARGs within respiratory pathogens at early time points could serve as a predictor of later phenotypic resistance, either broadly across drug classes or with respect to specific types of macrolide antimicrobials. The decision to consider ARGs detected within reads of *M. haemolytica*, *P. multocida*, *H. somni*, or *B. trehalosi* collectively, rather than by individual bacteria, reflected the potential for horizontal gene transfer among these bacteria [38], as well as the understanding that antimicrobial use (AMU) for BRD typically targets the entire bacterial complex rather than individual species [39].

What distinguishes this study from other sequencing-based BRD investigations [40,41,42,43] is its use of long-read metagenomic sequencing to describe bacteria and ARGs near the time of arrival at the feedlot. Long-read sequencing data has an advantage over 16S or other short-read sequencing approaches because bacteria and their ARGs can be identified within the same individual read. To our knowledge, no prior studies have assessed whether the detection of specific bacteria with ARGs from long-read metagenomic sequencing in calves without clinical signs was associated with a later BRD risk or phenotypically expressed antimicrobial resistance at the time of treatment. Instead, previous studies have either (a) evaluated the real-time concordance between ARGs and phenotypic resistance of the same samples [35,44,45], (b) described the prevalence of ARGs in BRD cases or mortalities [46,47,48], (c) compared microbial populations between healthy and BRD-affected cattle [7,42,47,49,50,51,52], or (d) explored the associations between viruses and BRD [50,53].

The ability to report ARGs directly assigned to *Pasteurellaceae* reads improves clinical relevance by connecting resistance to key bacteria in a single step rather than simply reporting ARGs detected within the broader microbiome or using a multistep method of culture followed by PCR or whole genome sequencing for ARG detection. While traditional culture and AST provide AMR information on a limited number of pathogens under specific laboratory conditions, metagenomic sequencing offers the ability to simultaneously detect a broader range of pathogens and associated resistance determinants [22]. In this study, ARGs on *B. trehalosi* reads were considered in addition to the three BRD organisms where isolates were collected for AST.

Metagenomic sequencing near feedlot arrival can support diagnostic and surveillance objectives, promoting antimicrobial stewardship in beef production. Although ARG detection as a proxy for resistance is likely conservative, as not all resistance genes will be expressed, we observed that the detection of these genes is associated with a subsequent phenotypic resistance in animals treated for BRD. Performing laboratory diagnostics on individual cattle at the time of illness to inform precision treatment for that animal is not currently feasible at the commercial level [54]. Therefore, early metagenomic profiling in advance of clinical disease could be used to anticipate resistance risks. Incorporating such information into antimicrobial decision-making could decrease the use of drug classes for which resistance determinants are already present, thereby reducing the risk of treatment failure and limiting the selection pressure for resistant pathogens [19,55,56,57]. A recent study has proposed how this information could be integrated into feedlot decisions [58], with demonstrated interest from Canadian feedlot veterinarians [54].

While the logic of this approach has been recognized, clinical response depends on much more than just the in vitro susceptibility of bacteria [59]. Several factors play a role in the treatment efficacy, including animal health status, stage of infection, bacterial virulence, pathogen load, environmental stressors, acceptable error rates of MIC interpretation, sample handing, and how efficacy is defined [60,61,62,63,64,65,66]. Thus, the relationship between in vitro susceptibility and in vivo treatment response remains poorly defined in BRD [64,67]. Similar discussions are also occurring in human medicine due to the multifactorial nature of disease and host–pathogen dynamics [66,68,69,70]. This study did not aim to evaluate treatment outcomes directly, but rather to explore a means of improving antimicrobial decision-making from the current empirical standards. More work is required to fully evaluate the potential linkages between genotypic or phenotypic resistance and BRD incidence and treatment outcomes.

One of the key implications of this study was the importance of sample timing in predicting BRD and AMR risk. While the detection of bacteria carrying ARGs at 13 DOF was associated with AMR phenotypes observed at BRD treatment before 45 DOF, a detection at arrival processing was not. This mirrors culture-based findings in the same population, which demonstrated a limited phenotypic resistance at arrival processing [23], supporting the idea that AMR patterns might only become informative after the respiratory microbial community has shifted post-feedlot placement [27,43,48,71,72,73,74,75], since metaphylaxis, commingling, and the feedlot environment are known to drive ARG acquisition and bacterial transmission [76,77,78,79,80]. Consistent with the low ARG detection at 1 DOF, a low AMR prevalence has been reported in calves at weaning in Canadian cow–calf herds [81,82]. Together, these results suggest that ARG-based predictions might only become informative after the initial feedlot exposure.

The metagenomic detection of BRD-associated pathogen bacteria detected by metagenomic sequencing showed time- and scale-dependent associations with BRD risk. At the calf level, the detection of bacteria at 1 DOF was not significantly associated with future disease, parallelling the culture-based results [23]. However, at 13 DOF, *H. somni* detection was associated with a later BRD diagnosis of individual calves, while *M. haemolytica* and *P. multocida* were not, differing from culture-based findings where all three were associated [23]. For pen-level analyses, higher 13 DOF prevalences of *M. haemolytica* and *P. multocida* were associated with a higher risk of BRD in calves from those pens, an association not detected by the culture [23]. These findings highlight the potential added value of pen-level metagenomic surveillance in detecting broader transmission patterns.

While sequencing was performed on nearly all calves sampled at the time of BRD diagnosis (128/130 calves), the smaller number of longitudinally sampled calves limited the statistical power relative to the culture-based dataset [23]. Nonetheless, the observed associations trended in the same direction, and Bayesian latent class models suggested a comparable sensitivity of long-read metagenomic sequencing for detecting macrolide and tetracycline resistance genes to that of culture and AST, with a specificity exceeding 95% across both methods [30]. The categorization of intermediate MICs influenced the strength, but not direction, of associations. The detection of *mphE-msrE* was consistently associated with non-susceptibility to 15-membered macrolides regardless of MIC categorization. However, the categorization of intermediate MIC results influenced the strength of associations observed for *EstT* and *tet(H)*. Nevertheless, the direction of association remained consistent, and the presence of *EstT* or *tet(H)* at 13 DOF continued to trend towards an increased likelihood of future resistance.

Pen-level findings were more robust across categorization schemes. Specifically, a higher pen-level prevalence of *mphE-msrE* was consistently associated with the detection of phenotypic resistance to 15-membered macrolides at the time of BRD diagnosis, while *EstT* prevalence was consistently associated with a resistance to 16-membered macrolides. These pen-level metagenomic findings directly support the previous pen-level AST-based associations between macrolide and tetracycline resistance at 13 DOF and subsequent AMR at BRD treatment [23]. They further underscore the influence of shared environments, microbial transmission, and collective ARG exposure in shaping AMR outcomes [24,29,83] and the potential of pen-level data for predicting AMR risk without the need for tracking individual calves.

The imperfect concordance between ARG detection and phenotypic resistance reflects both technical limitations and biological complexity. Culture and AST are constrained by bacterial growth requirements, MIC reproducibility, and breakpoint interpretation [84,85]. ARG expression is influenced by regulatory mechanisms [86], gene amplification and copy number [87], genomic context [88], and the presence of other resistance mechanisms, many of which remain poorly characterized in BRD pathogens. For example, prior studies have shown inconsistent associations between *EstT* and macrolide subclasses [36]. Bayesian models similarly found that *mphE-msrE* and *EstT* were not uniquely predictive of 15- versus 16-membered macrolide phenotypic resistance [30]. Additional limitations include the low baseline prevalence of ARGs and phenotypic resistance at arrival in the present study [23], and the resulting focus on only macrolides and tetracyclines.

This study was unique in exploring the predictive value of *B. trehalosi* detection and subsequent BRD risk, beyond simply describing recent recovery in association with the disease [13,89,90]. However, *B. trehalosi* was relatively uncommon in the sequence data as compared to the other *Pasteurellaceae* of interest, and no comparator diagnostic data (e.g., culture or PCR) were available to inform a detection threshold as was performed for the other bacteria. As such, a sample was classified as positive if the theoretical coverage for *B. trehalosi* exceeded the 80th percentile of coverage values for that study year. This represents a methodological limitation when compared to the data-driven cutoff selection used for the other organisms in this study.

While *Mycoplasmopsis bovis* has been recognized as an important contributor to BRD [91,92,93] and was identified by long-read metagenomic sequencing [5,22], it was not targeted for analysis in the present study. The focus of the present study was on AMR associated with ARGs in *Pasteurellaceae* bacteria. In contrast, AMR in *M. bovis* is primarily mediated by mutations, and susceptibility studies typically require whole genome sequencing data or phenotypic data [94,95,96].

The potential for selection bias must be considered in the present study, given that metagenomic sequencing data were not available from all calves. While samples from at least half of the calves in each pen were subjected to sequencing, most samples were not randomly selected. Rather, the samples sequenced at arrival and 13 DOF were initially chosen to allow for optimizing the potential for longitudinal data analyses, with the remainder of the sample randomly selected in matched arrival/13 DOF pairs to target at least 50 calves per pen. This strategy was necessary for the longitudinal subset analysis reported here comparing the detection of ARGs at arrival and 13 DOF with AMR at the time of treatment. With respect to using the larger set of the same samples to examine associations between the detection of organisms and a later risk of BRD treatment, again, this strategy ensured that the treated animals were represented in the available data. However, in both years, there were more calves sampled at 36 DOF [30] and targeted in the arrival and 13 DOF samples than sick calves, ensuring that the sequencing data was not limited to prior samples from sick calves.

Resistance outcomes were assessed only within the first 45 DOF, the period when most BRD cases occur [97]. Without metaphylaxis, BRD often occurs within the first two weeks on feed [97,98]. However, with metaphylaxis, particularly with long-acting macrolides such as tulathromycin, which can have a post-metaphylactic interval of up to 14 days [99], onset is frequently delayed. Consistent with other reports [100,101], most BRD cases in tulathromycin-treated cohorts from the present study occurred after the first two weeks on feed [23], supporting the relevance of sampling at 13 DOF. However, given that the respiratory microbiome continues to shift throughout the feeding period, additional sampling later in production would be necessary to assess the resistance risk during the mid-to-late phases of feeding. Future research should also assess whether similar associations hold across other antimicrobial classes or in cattle with different risk profiles (e.g., high-risk newly received calves vs. low-risk backgrounded cattle).

One other potential source of bias in the present study was the potential for barcode crosstalk or the misclassification of sample results within a given library to the wrong barcode. Modifications were made to the library preparation to minimize this risk, as described in the study methods. While there was some evidence of the detection of high prevalence bacteria in raw read data, suggesting some residual barcode crosstalk, the evidence-informed BLCM cutoffs mitigated the potential for a substantial misclassification, as any counts in negative control samples were below threshold values [30]. Furthermore, there was very limited to no evidence of ARG detection in the negative controls (1 sample in 2020 and 0 in 2021). Finally, data from the comparison to the culture reported in a parallel Bayesian latent class analysis supported the specificity of sample classification based on at least one raw read where the ARG of interest was identified on a read also classified as a target bacterial species [30].

More broadly, the lack of methodological standardization in metagenomics—including the variation in sequencing platforms, bioinformatic pipelines, and detection thresholds—currently hampers comparability across studies [102,103,104]. The continued development of benchmarks, along with reductions in cost and turnaround time, will be critical for translation to field settings. Ongoing attention to the reporting and sharing of metagenomic data [105] will further support the validation of genomic tools for informing antimicrobial stewardship. The use and application of pen-level metagenomic testing to inform antimicrobial decisions in commercial feedlots have also been investigated using simulation tools [106], but additional studies are needed to evaluate any risks associated with sampling cattle at 13 DOF.

## 4. Methods

The core metagenomic sequencing data from the fall-placed calves reported in this study, including detailed descriptions of bacterial and ARG detection across time points, are reported elsewhere [30]. The present analysis builds on those findings by evaluating associations with BRD outcomes and phenotypic resistance.

### 4.1. Ethics Statement

This study was conducted in accordance with the recommendations of the Canadian Council of Animal Care (CCAC) [107]. The research protocols and procedures for this study were approved on 30 May 2019 by the University of Saskatchewan Animal Care Committee (AUP 20190069) and reviewed annually until study completion.

### 4.2. Study Population

The calves included in this study were also part of a broader investigation evaluating changes in the prevalence of cultured *M. haemolytica*, *P. multocida*, and *H. somni*, along with AST results in feedlot calves at two early feeding period time points [27]. Comprehensive details regarding the study population, full bacterial culture, and AST data for all sampled calves at 1 DOF and 13 DOF have been described [27].

Auction-sourced, mixed-origin steer calves (*n* = 1600) were enrolled over two years, with one animal removed for lameness, leaving 1599 calves. Calves were purchased in groups of 100 over an eight-week period from late September to mid-November in both 2020 and 2021 (800 calves per year). They then remained with their arrival cohorts in pens of 100 until 45 DOF. Monitoring and treatment protocols for BRD, as well as culture results and factors associated with BRD in these calves, were also previously described [23].

The mean arrival bodyweight (BW) in 2020 was 253 kg (556 lbs) [range: 211–291 kg (464–640 lbs), standard deviation: 14 kg (31 lbs)]. In 2021, the mean BW was lighter: 225 kg (496 lbs) [range: 160–315 kg (351–694 lbs), standard deviation: 15 kg (34 lbs)]. Pens included calves derived from 30 to 81 unique farms of origin in 2020 and 12 to 38 in 2021. The study facility consisted of eight outdoor earthen-floor pens, each accommodating up to 100 calves and designed according to Canadian feedlot cattle housing guidelines [108]. Pens were arranged in two groups of four, separated by a building, with adjacent pens sharing fence-line watering bowls. Calves were provided a diet formulated to meet or exceed National Research Council nutritional requirements for beef cattle [109].

### 4.3. Processing and Sample Collection

Calves were processed the morning after arrival (1 DOF) following standard protocols used for moderate- to high-risk cattle in commercial feedlot settings. All calves arriving in 2020 (*n* = 800 calves, Pens 1–8) received parenteral tulathromycin (Draxxin^®^, Zoetis Inc., Florham Park, NJ, USA; 2.5 mg/kg BW) as metaphylaxis upon arrival. In 2021, metaphylaxis protocols were split, with half of pens (*n* = 400 calves, Pens 9–11, 16) receiving injectable oxytetracycline (Oxyvet^®^ 200 LA, Vetoquinol, Lavaltrie, QC, Canada; 20 mg/kg BW) and the other half (*n* = 400, Pens 12–15) receiving tulathromycin.

Deep nasopharyngeal swabs (DNPSs) were collected at arrival processing (1 DOF) before metaphylaxis administration and at 13 DOF. A subset of calves from each pen were sampled at 36 DOF (*n* = 310). Three DNPSs were obtained from alternating nostrils and pooled into a 15 mL vial containing 3 mL of liquid Amies transport medium (CoPan Diagnostics, Carlsbad, CA, USA).

### 4.4. Calves Receiving First Treatment for BRD

Experienced feedlot personnel conducted daily health assessments to identify calves displaying clinical signs of BRD. A standardized DART scoring system (Depression, Appetite, Respiratory signs, and Temperature) was used to evaluate affected animals [23]. Clinical severity was graded on a numerical scale from 0 (normal) to 4 (moribund). Calves were treated for BRD if they exhibited a score of 1 or 2 and were febrile (rectal temperature ≥ 40°C), or if they had a score of 3 or 4 regardless of temperature and provided no other underlying cause of illness was identified [83].

DNPS were collected from calves meeting the BRD case definition prior to antimicrobial treatment and then stored in a refrigerator (4 °C for ≤72 h) until transport to the laboratory. 

### 4.5. Microbiology Methods and Isolate Selection

The laboratory microbiology procedures have been previously described [27]. Samples collected at 1 DOF and 13 DOF were batch processed on the day of collection and samples from treated calves the day of arrival at the lab. Samples were vortexed for 1 min, and a 300 μL aliquot, pooled from the three collected swabs, was submitted to Prairie Diagnostic Services, Inc. (PDS), Saskatoon, SK, Canada for culture and AST.

For bacterial culture, 10 μL of the pooled sample was inoculated onto Columbia agar with sheep blood and chocolate agar (Oxoid, Thermo Fisher, Waltham, MA, USA) and incubated at 35 °C for 18 h in a 5% CO_2_ atmosphere. Speciation was confirmed using matrix-assisted laser desorption/ionization time-of-flight mass spectrometry (MALDI-TOF MS) using a Microflex LT instrument (Bruker Daltonik, Bremen, Germany) and Biotyper Microflex LT Compass version 1.4 software (Bruker Corporation, Billerica, MA, USA). Only colonies with MALDI-TOF MS identification scores ≥ 2.0 were included in further analysis. Positive and negative controls were tested on all sample processing days and new media batches. The positive controls were *Staphylococcus aureus* ATCC 29213, *Escherichia coli* ATCC 25922, and *Histophilus somni* ATCC 700025. A plain matrix spot was run with every MALDI run to ensure no contamination.

Antimicrobial susceptibility testing (AST) was completed using a commercially available bovine serial broth microdilution panel (Vet Bovine AST BOPO7F Plate, Thermo Fisher Scientific™, Mississauga, ON, Canada) on the Sensititre™ platform. Quality control measures were performed according to the manufacturer’s guidelines using *E. coli* ATCC 25922, *S. aureus* ATCC 29213, and *H. somni* ATCC 700025. Minimum Inhibitory Concentration (MIC) plates were processed and interpreted using a BIOMIC^®^ V3 microplate reader (Giles Scientific Inc., Santa Barbara, CA, USA). MIC values for each antimicrobial were compared against Clinical and Laboratory Standards Institute (CLSI) breakpoints, except for tilmicosin, for which breakpoints are only available for *M. haemolytica* [60].

### 4.6. Long-Read Metagenomic Sequencing and Classification of Positive Results

#### 4.6.1. Metagenomic Sequencing Sample Preparation Protocols

A subset of samples was sequenced for this study. Samples were selected for metagenomic sequencing to optimize use of the longitudinal calf information collected from study samples and to allow for sequencing of at least half of the calves from each pen. Initially, all calves that received treatment for BRD or from the subset of calves sampled at day 36 were identified. They and their matching samples from 1 and 13 DOF were selected for sequencing from each pen. Additional calf samples from 13 DOF with matching samples at 1 DOF were then randomly selected with a goal of at least 50 calves per pen.

#### 4.6.2. 2020 Sample Processing Protocol

The DNPSs collected in the fall of 2020 were stored at −80 °C after aliquoting for culture and AST. The stored DNPSs were later processed for metagenomic sequencing using a modified protocol [22]. For the initial sample enrichment step, the swab heads were placed into glass vials with stir bars capped with air permeable membranes (Thomson, Carlsbad, CA, USA) with 7–10 mL of brain heart infusion broth (BHI) (Oxoid, Thermo Fisher, Waltham, MA, USA) + 1% glucose. Vials were aerated and incubated at 35–37 °C for 14 h. Enriched media were collected for downstream DNA extraction.

An aliquot (1.5 mL) of each enriched sample was pelleted at 4000 rpm × 10 min and then resuspended in 100 μL 1× phosphate-buffered saline (PBS). To host deplete, the sample was treated with 1× DNase (Invitrogen, Waltham, MA, USA) for 30 min at 37 °C × 300 rpm. DNase was inactivated via heat treatment at 75 °C for 10 min × 300 rpm. Samples were pelleted by centrifugation at 4000 rpm × 10 min and washed by resuspension in 100 μL with 1× PBS for DNase removal. Cells were pelleted by centrifugation at 4000 rpm × 10 min, and the wash buffer was removed.

DNA extraction was completed via alcohol precipitation, using the Qiagen Puregene Buccal Cell Kit (QIAGEN Inc., Germantown, MD, USA). Pelleted cells were washed and resuspended in Qiagen Puregene Cell Lysis Buffer (QIAGEN Inc., Germantown, MD, USA), then treated with proteinase K (QIAGEN Inc., Germantown, MD, USA) at 55 °C for 1 h at 300 rpm. Following protein digestion, samples were treated with RNase A (QIAGEN Inc., Germantown, MD, USA) at 37 °C for 15 min at 300 rpm. Liberated proteins were precipitated via Qiagen Puregene Protein Precipitation Solution (QIAGEN Inc., Germantown, MD, USA) and then cooled on ice until proteins had coagulated and could be pelleted by centrifugation.

Supernatants were carefully removed to fresh tubes to avoid protein contamination. Nucleic acids were precipitated using isopropanol with glycogen (QIAGEN Inc., Germantown, MD, USA) as a carrier, incubated at room temperature for 5 min to ensure maximum DNA retention, then spun for 5 min at max speed (14,000 to 16,000 rpm). Pellets were washed with 70% ethanol, and the wash was removed, and the pellet was allowed to air dry. The DNA pellet was resuspended in Qiagen Puregene DNA Hydration Solution (QIAGEN Inc., Germantown, MD, USA) for 1 h at 65 °C and then overnight by gentle rotation at room temperature. Extracted DNA was stored at 4 °C until sequencing.

A minimum of 2 μg DNA (50 ng/μL in 40 μL) was used for library preparation. Samples were tested using Qubit 1 × dsDNA HS Assay Kit (Invitrogen, Waltham, MA, USA); 394 samples were re-extracted from reserve enriched aliquots to obtain 2 μg DNA.

#### 4.6.3. 2021 Sample Processing Protocol

The DNPSs collected in the fall of 2021 were processed immediately after collection and aliquoted for culture and AST. Fresh swabs and remaining media were incubated in BHI + 1% glucose at 35–37 °C as described for the 2020 samples, but for 10 h. The 2021 samples did not have a DNase treatment to remove free DNA prior to pelleting, as pilot testing had indicated it was unnecessary to achieve adequate sequencing results on samples processed without prior freezing [22]. Sample DNA was extracted and processed as for the 2020 samples. Extracted DNA was stored at 4 °C until sequencing.

#### 4.6.4. 2020 and 2021 Library Preparation and Sequencing Protocol

The 2021 samples, prepared from fresh swabs and DNA, were sequenced first, followed by the 2020 samples that were prepared from frozen swabs. Extracted DNA was purified and size selected by a 0.4 × cleanup with AMPure XP beads (Beckman Coulter, Indianapolis, IN, USA). Normalization to 400 ng was performed (where sufficient DNA was present, or 200 ng if not) prior to library preparation. For the samples with insufficient DNA concentration after size selection, non-size-selected DNA was used.

Library preparation was completed following the Oxford Nanopore Technologies (ONT) ligation protocol with native barcoding (SQK-LSK109 and EXP-NBD196, Oxford Nanopore Technologies, Oxford, UK) in a 96-well plate high-throughput library format with minor modifications to minimize barcode crossover or background barcode crosstalk [110]. Barcode ligation was followed by the addition of 1 µL of EDTA (Invitrogen, Waltham, MA, USA), a 10 min room temperature incubation, and a 10 min 65 °C incubation. DNA from barcoded samples was pooled into 38 libraries for the 2021 samples and 36 libraries for the 2020 samples, with each library containing up to three negative controls (nuclease free water, Invitrogen, Waltham, MA, USA).

From each prepared library, 80–200 ng (2021 samples) and 70–150 ng (2020 samples) were delivered to the Omics and Precision Agriculture Laboratory (OPAL, University of Saskatchewan, Saskatoon, SK, Cananda) for ONT sequencing with the PromethION24 platform. Libraries containing sample pools were loaded onto FLO-PRO002 flow cells (ONT version R9.4.1, Oxford Nanopore Technologies, Oxford, UK) according to standard procedures, except for using molecular grade H_2_O (Sigma-Aldrich, St. Louis, MO, USA, or Promega, Madison, WI, USA) in place of loading beads to reduce pore saturation during sequencing.

Sequencing was carried out with standard default run parameters using high accuracy base calling with a Q-score cut-off of 7. Sequencing runs were active for 72 h for the 2021 samples and 48 h for the 2020 samples. The change from 72 h (2021 samples) to 48 h (2020 samples) followed a series of retrospective analyses on 2021 sequence data to assess the rates of data acquisition and identified that sequencing beyond 48 h did not alter sample interpretation.

#### 4.6.5. Preprocessing/Quality Control

Data from MinKNOW were further processed for quality control with Porechop (version 0.2.4) [111] and Nanofilt (version 2.8.0) [112] to remove adapters and short (<200 bp) reads. NanoStat (version 1.6.0) provided statistics about the distribution of read length by total base pairs per sample [112].

#### 4.6.6. Read Classification and Host Filtering

The taxonomic classification of reads was achieved using Kraken 2 (version 2.1.2) [113]. A custom database was used for Kraken 2 classification, which included bacterial, viral, and archaeal subsets of the November 2023 RefSeq database [114] as well as the *Bos taurus* ARS-UCD1.2_Btau5.0.1Y genome assembly accessed on 30 September 2025 and available at https://sites.ualberta.ca/~stothard/1000_bull_genomes/ [5,115]. Typically, sequences classified as host would be removed before downstream processing; however, a small population of chimeric *B. taurus* bacterial reads (<0.1% of all reads) was detected. A custom program, kmer_filter.py, was written to retrieve host-classified reads that met a threshold of 25% non-host sequence using Kraken 2 k-mer identity and included these as potential non-hosts for downstream processing. The rationale behind this step was to cast the widest possible net for ARG detection, even if a small amount of host sequence remained.

Host-filtered reads (i.e., those not similar to *B. taurus* taxid 9913) were extracted using the KrakenTools (version 1.2) [116] utility extract_kraken_reads.py, and these were added to the chimeric reads using a combination of Bash utilities and the BBTools (version 38.86) [117] filterbyname.sh script. Bracken (version 2.7) [118] with a minimum read length of 200 bp (“--read-length 200”) was used to improve the species-level estimation of abundance reported by Kraken 2. Reads classified as hosts were removed from further consideration. A custom script, report_taxon_read_lengths.py, added additional context to the Bracken results, including the total amount of sequence in base pairs reported for each species (including child taxa) and the fraction of total classified sequence.

#### 4.6.7. Antimicrobial Resistance Gene Detection

To identify reads containing ARGs, non-host reads were first converted to FASTA format using Seqtk (version 1.3) [119]. ARGs were identified in non-host reads using Abricate (version 1.0.0) [120] and AMRFinderPlus (version 3.11.18) [86], both with the NCBI Bacterial Antimicrobial Resistance Reference Gene Database (version 2023-11-15.1) and the parameters “--plus --coverage_min 0.8 --ident_min 0.8” to identify the plus genes while requiring a minimum coverage and minimum identity of 80%. Abricate was also run using the Comprehensive Antimicrobial Resistance Database (CARD) (version 3.2.8) [121]. For AMRFinderPlus, the minimum percent identity and percent coverage thresholds were set to 80%, and the -plus option was used to direct the program to search for genes involved in virulence, biocide, heat, metal, and acid resistance. Default parameters were used for Abricate (80% minimum percent identity and percent coverage). ARG results from NCBI and CARD were merged based on gene name and start/stop coordinates. Once merged, the CARD gene names were preferentially retained in downstream reports.

#### 4.6.8. Classification of Metagenomic Results as Positive or Negative

As previously reported [30], cutoffs based on a minimal threshold of reported theoretical coverage for each organism were used to categorize metagenomic sample data as positive or negative for the detection of *M. haemolytica*, *P. multocida*, and *H. somni*. Theoretical coverage for each sample was calculated by dividing the total read length for each bacterium by the size of its reference genome (*M. haemolytica*: 2.8 Mb [NCBI GCF_002285575.1], *P. multocida*: 2.3 Mb [NCBI GCF_002073255.2], *H. somni*: 2.3 Mb [NCBI GCF_000019405.1]). Theoretical coverage was then evaluated against culture results using receiver operating characteristic curves in R (pROC) [122] to determine a baseline cutoff based on maximizing the combined sensitivity and specificity (Youden’s index) of metagenomics compared to culture. If the initial BLCM resulted in a specificity of <0.9, the theoretical coverage cutoff was increased until a specificity of ≥0.9 was obtained. The final cutoffs used for classification of each organism, for 2020 and 2021, respectively, were as follows: *M. haemolytica* (5.1×, 1.7×), *P. multocida* (1.2×, 0.26×), and *H. somni* (0.09×, 0.05×).

No culture data were available for *B. trehalosi*; therefore, a more arbitrary cutoff was necessary. A sample was classified as positive if the theoretical coverage calculated based on the *B. trehalosi* reference genome (NCBI GCF_000521725.1) (2.3 Mb) was greater than the 80th percentile for coverage in each year. This reflected a conservative estimate based on comparison to the deciles corresponding to the BCLM-determined cutoffs for the other bacteria, which typically fell below the 80th percentile. The resultant cutoffs for *B. trehalosi* were 0.19× for 2020 and 0.02× for 2021.

For long-read metagenomic sequencing detection of ARGs, a positive result was defined as a DNP sample where there was detection of any relevant ARGs (*mphE-msrE*, *EstT*, or *tet(H))* within at least one read assigned to *M. haemolytica*, *P. multocida*, *H. somni*, or *B. trehalosi*. The identification of *mphE* and *msrE* was considered in combination and defined as “either/or” or “both,” since these genes are typically arranged in tandem and co-expressed from the same promoter [31]. 

### 4.7. Statistical Analysis

Data were managed in a commercial spreadsheet program (Microsoft Excel, version 2401, Microsoft Corporation, Redmond, WA, USA). Analyses were completed using the Stata^®^ statistical software package (Stata/IC, version 16.1, StataCorp LLC, College Station, TX, USA) unless otherwise noted.

Two primary study questions evaluated the relationship between long-read metagenomic sequencing detection of BRD-associated bacteria and ARGs at and shortly after arrival with the odds of subsequent BRD treatment and antimicrobial susceptibility outcomes at the time of BRD treatment. The regression models examined in this study are summarized in Table 4 and detailed as follows. At the calf level, the primary outcomes of interest included (1) the likelihood of a calf being treated for BRD and (2) culture and AST results at the time of first BRD treatment. Table 4 provides a summary of the primary outcomes analyzed based on the timing of BRD treatment and the sequencing-based data examined as potential risk factors for each analysis.

The first set of models focused on whether metagenomic sequencing detection of BRD-associated *Pasteurellaceae* species (*M. haemolytica*, *P. multocida*, *H. somni*, or *B. trehalosi*) at 1 DOF or 13 DOF were associated with the subsequent risk of treatment for BRD (Table 4). Specifically, the models assessed whether detection of bacteria by sequencing (detected/not detected based on thresholds determined by ROC and BLCM models described previously) for an individual calf at 1 DOF was associated with the likelihood of BRD treatment on or before 13 DOF for that calf. Similarly, the models assessed whether sequencing results at 13 DOF were associated with BRD treatment reported between 14 DOF and 45 DOF.

The second set of models focused on the subset of calves that received BRD treatment (*n* = 128 calves for which sequencing data were available) and examined whether the sequencing-based detection of ARGs in BRD-associated bacteria at 1 DOF or 13 DOF was associated with the likelihood of specific AST results at the time of first BRD treatment (Table 4). The specific ARGs and corresponding phenotypic outcomes considered in these models included *msrE-mphE* identified near arrival associated with phenotypic resistance at the time of BRD treatment to (a) any macrolide for which AST results and CLSI breakpoints were available (gamithromycin, tulathromycin, tildipirosin, and/or tilmicosin), (b) phenotypic resistance to the 15-membered ring macrolides (tulathromycin or gamithromycin), and (c) phenotypic resistance to the 16-membered ring macrolides (tildipirosin or tilmicosin). The detection of *EstT* was also evaluated for associations with any macrolide, 15-membered ring macrolides, or 16-membered ring macrolides. Lastly, the detection of *tet(H)* was evaluated for its association with phenotypic resistance to tetracyclines.

As in the previous models, sequencing detection of each ARG within a read assigned to the *Pasteurellaceae* species of interest was treated as a binary variable (detected vs. not detected). For AST data, isolates classified as “intermediate” based on MIC values were dichotomized in two ways: (a) categorized as susceptible, resulting in a binary outcome of *susceptible (including intermediate)* vs. *resistant*, and (b) categorized as resistant, resulting in a binary outcome of *susceptible* vs. *non-susceptible (including intermediate and resistant)*. Both categorizations were compared to evaluate how interpretive differences impacted associations with ARGs.

All sets of models where at least one *p*-value was significant were repeated as described above, but where the risk factor of interest was the pen-level prevalence of detection of either the bacteria of interest or the ARG of interest, as appropriate, rather than the value for the individual calf. Pen-level prevalence was scaled to reflect 10% increments to generate odds ratios within an interpretable range. All significant associations were examined for linearity by adding the square of the prevalence to the model and testing it for significance.

## 5. Conclusions

In summary, the long-read metagenomic sequencing of DNPS samples collected from feedlot calves near two weeks on feed can provide valuable insights into AMR potential prior to BRD diagnosis within 45 DOF. At the individual calf level, the presence of key ARGs within *Pasteurellaceae*-assigned reads at 13 DOF were significantly associated with a corresponding phenotypic resistance at the time of BRD diagnosis later in the feeding period. Specifically, *mphE-msrE* and *EstT* were associated with a subsequent macrolide resistance and *tet(H)* with tetracycline resistance. At the pen level, higher prevalences of *mphE-msrE* or *EstT* within pens at 13 DOF were similarly associated with a greater likelihood that a calf tested from the same pen would harbor bacteria resistant to 15- and 16-membered ring macrolides at the time of BRD diagnosis. The deployment of these methods across diagnostic laboratories will depend on factors such as turn-around time, cost, and standardization across laboratories. These findings offer preliminary evidence that long-read sequencing at this early post-arrival time point could support targeted, timely, and responsible antimicrobial treatment decisions in feedlot cattle.

## Figures and Tables

**Table 1:** (**a**) Calf-level risk of subsequent treatment for bovine respiratory disease (BRD) given metagenomic long-read sequencing detection of BRD bacteria from deep nasal pharyngeal swab samples collected at arrival processing (1 day on feed (DOF)) or 13 DOF. (**b**) Calf-level risk of subsequent treatment for BRD given 10% increase in pen-level metagenomic long-read sequencing detection of BRD bacteria from deep nasal pharyngeal swab samples collected at arrival processing (1 DOF) or 13 DOF.

(a)				
Risk Factor: Sequencing Detection of Bacteria	Odds Ratios	95% Confidence Interval		*p*-value
		Lower	Upper	
Metagenomic detection at 1 DOF (*n* = 840 calves)	Outcome: BRD ≤ 13 DOF			
*M. haemolytica*	0.97	0.58	1.6	0.90
*P. multocida*	0.87	0.49	1.5	0.63
*H. somni*	0.86	0.27	2.7	0.80
*B. trehalosi*	0.86	0.27	2.7	0.80
Metagenomic detection at 13 DOF (*n* = 819 calves)	Outcome: BRD 14 to 45 DOF			
*M. haemolytica*	1.4	0.95	2.1	0.09
*P. multocida*	1.8	0.78	4.2	0.17
*H. somni*	2.1	1.04	4.3	**0.04**
*B. trehalosi*	0.70	0.44	1.1	0.15
(b)				
Risk Factor: Sequencing Detection of Bacteria	Odds Ratios	95% Confidence Intervals		*p*-value
		Lower	Upper	
Pen-level metagenomic detection at 13 DOF (10% increase) (*n* = 819)	Outcome: BRD 14 to 45 DOF			
*M. haemolytica*	1.16	1.00	1.34	**0.04**
*P. multocida*	1.14	1.01	1.27	**0.03**
*H. somni*	1.16	0.82	1.66	0.41
*B. trehalosi*	1.00	0.81	1.22	0.97

Models account for year and metaphylaxis and adjust for clustering by pen (*N* = 16 pens). Statistically significant associations (*p* < 0.05) are bolded in the text.

**Table 2:** For calves treated for bovine respiratory disease (BRD) at or before 13 days on feed (DOF) (*n* = 64 calves), associations between antimicrobial resistance genes (ARGs; *mphE-msrE*, *EstT*, or *tet(H)*) within bacterial reads of *M. haemolytica, P. multocida*, *H. somni*, or *B. trehalosi* detected from deep nasopharyngeal swabs collected from calves at arrival processing (1 DOF), and the likelihood of corresponding phenotypic antimicrobial susceptibility testing (AST) results at the time of first treatment for BRD.

(i) Dichotomous Outcome: Susceptible + Intermediate vs. Resistant					
Risk Factor: ARGs detected in *Pasteurellaceae* bacteria at 1 DOF	Outcome: Resistant AST in *Pasteurellaceae* bacteria at BRD treatment ≤ 13 DOF	Odds ratio	95% confidence intervals		*p*-value
			Lower	Upper	
*msrE-mphE*	Tula/Gam/Tild/Tilm	no calves with *mphE-msrE* at 1DOF			
*msrE-mphE*	Tula/Gam				
*msrE-mphE*	Tild/Tilm				
*EstT* **	Tula/Gam/Tild/Tilm	31	0.0001	1209	>0.99
*EstT* **	Tula/Gam	31	0.0001	1209	>0.99
*EstT*	Tild/Tilm	no calves with Tild/Tilm at 1 DOF			
*tet(H)* **	Tet	7.8	0.40	152	0.18
(ii) Dichotomous Outcome: Susceptible vs. Intermediate + Resistant (Non-susceptible)					
Risk Factor: ARGs detected in *Pasteurellaceae* bacteria at 1 DOF	Outcome: AST in *Pasteurellaceae* bacteria at BRD treatment ≤ 13 DOF	Odds ratio	95% confidence intervals		*p*-value
			Lower	Upper	
*msrE-mphE*	Tula/Gam/Tild/Tilm	no calves with *mphE-msrE* at 1 DOF			
*msrE-mphE*	Tula/Gam				
*msrE-mphE*	Tild/Tilm				
*EstT* **	Tula/Gam/Tild/Tilm	9.7	0.0001	377	>0.99
*EstT* **	Tula/Gam	20	0.0001	793	>0.99
*EstT* **	Tild/Tilm	12	0.0001	460	>0.99
*tet(H)* **	Tet	7.1	0.35	144	0.20

AST data were not available for *B. trehalosi*. Tula, tulathromycin (15-membered ring macrolide); Gam, gamithromycin (15-membered ring macrolide); Tild, tildipirosin (16-membered ring macrolide); Tilm, tilmicosin (16-membered ring macrolide); Tet, tetracycline. Analysis accounted for year and injectable metaphylaxis administered and adjusted for clustering by pen (*N* = 16 pens). No statistically significant associations (*p* < 0.05) were reported. ** Median unbiased estimate, exact logistic regression.

**Table 3:** (**a**) For calves treated for bovine respiratory disease (BRD) between 14 and 45 days on feed (DOF) (*n* = 64 calves), associations between antimicrobial resistance genes (ARGs; *mphE-msrE*, *EstT*, or *tet(H)*) within bacterial reads of *M. haemolytica*, *P. multocida*, *H. somni*, or *B. trehalosi* detected from deep nasopharyngeal swab samples collected from calves at 13 DOF, and the likelihood of corresponding phenotypic antimicrobial susceptibility testing (AST) results at the time of first treatment for BRD. (**b**) For calves treated forBRD between 14 and 45 DOF (*n* = 64 calves), associations between 10% increase in pen-level detected prevalence of ARGs (*mphE-msrE*, *EstT*, or *tet(H)*) within bacterial reads of *M. haemolytica*, *P. multocida*, *H. somni*, or *B. trehalosi* from deep nasopharyngeal swab samples collected from calves at 13 DOF, and the likelihood of corresponding phenotypic AST results at the time of first treatment for BRD.

(a)					
(i) Dichotomous Outcome: Susceptible + Intermediate vs. Resistant					
Risk Factor: ARGs detected in *Pasteurellaceae* bacteria at 13 DOF	Outcome: AST in *Pasteurellaceae* bacteria at BRD treatment 14 to 45 DOF	Odds ratio	95% confidence intervals		*p*-value
			Lower	Upper	
*msrE-mphE* *	Tula/Gam/Tild/Tilm	27	1.7	427	**0.02**
*msrE-mphE* *	Tula/Gam	27	1.7	427	**0.02**
*msrE-mphE* *	Tild/Tilm	3.6	0.34	38	0.29
*EstT* *	Tula/Gam/Tild/Tilm	6.3	2.1	19	**0.001**
*EstT*	Tula/Gam	6.3	2.1	19	**0.001**
*EstT* *	Tild/Tilm	7.4	1.5	38	**0.02**
*tet(H)* **	Tet	11	2.04	73	**0.004**
(ii) Dichotomous Outcome: Susceptible vs. Intermediate + Resistant (Non-susceptible)					
Risk Factor: ARGs detected in *Pasteurellaceae* bacteria at 13 DOF	Outcome: AST in *Pasteurellaceae* bacteria at BRD treatment 14 to 45 DOF	Odds ratio	95% confidence intervals		*p*-value
			Lower	Upper	
*msrE-mphE*	Tula/Gam/Tild/Tilm	6.1	0.31	120	0.24
*msrE-mphE* *	Tula/Gam	18	1.3	258	**0.03**
*msrE-mphE*	Tild/Tilm	2.0	0.16	23	0.59
*EstT*	Tula/Gam/Tild/Tilm	14	2.5	78	**0.003**
*EstT* *	Tula/Gam	3.6	1.4	9.7	**0.01**
*EstT*	Tild/Tilm	3.3	0.37	30	0.28
*tet(H)* **	Tet	1.3	0.19	9.6	0.78
(b)					
(i) Dichotomous Outcome: Susceptible + Intermediate vs. Resistant					
Risk Factor: Pen-level ARG prevalence in *Pasteurellaceae* bacteria at 13 DOF (10% increase)	Outcome: AST in *Pasteurellaceae* bacteria at BRD treatment 14 to 45 DOF	Odds ratio	95% confidence intervals		*p*-value
			Lower	Upper	
*msrE-mphE* *	Tula/Gam/Tild/Tilm	2.00	1.13	3.53	**0.02**
*msrE-mphE* *	Tula/Gam	2.00	1.13	3.53	**0.02**
*msrE-mphE* *	Tild/Tilm	1.46	0.81	2.62	0.21
*EstT* *	Tula/Gam/Tild/Tilm	1.59	0.60	4.20	0.35
*EstT*	Tula/Gam	1.59	0.60	4.20	0.35
*EstT* *	Tild/Tilm	3.33	1.36	8.17	**0.01**
*tet(H)* **	Tet	7.37	0.23	234	0.26
(ii) Dichotomous Outcome: Susceptible vs. Intermediate + Resistant (Non-susceptible)					
Risk Factor: Pen-level ARG prevalence in *Pasteurellaceae* bacteria at 13 DOF (10% increase)	Outcome: AST in *Pasteurellaceae* bacteria at BRD treatment 14 to 45 DOF	Odds ratio	95% confidence intervals		*p*-value
			Lower	Upper	
*msrE-mphE*	Tula/Gam/Tild/Tilm	1.99	0.82	4.84	0.13
*msrE-mphE* *	Tula/Gam	1.83	1.10	3.06	**0.02**
*msrE-mphE*	Tild/Tilm	1.37	0.61	3.06	0.44
*EstT*	Tula/Gam/Tild/Tilm	2.57	1.02	6.49	**0.046**
*EstT* *	Tula/Gam	1.94	0.85	4.46	0.12
*EstT*	Tild/Tilm	3.83	1.43	10.2	**0.01**
*tet(H)* **	Tet	9.37	0.55	160	0.12

AST data were not available for *B. trehalosi*. Tula, tulathromycin (15-membered ring macrolide); Gam, gamithromycin (15-membered ring macrolide); Tild, tildipirosin (16-membered ring macrolide); Tilm, tilmicosin (16-membered ring macrolide); Tet, tetracycline. Dichotomous AST outcomes are displayed with “intermediate” minimum inhibitory concentration (MIC) values categorized in two ways: (a) as susceptible, yielding a binary outcome of susceptible (including intermediate) vs. resistant; and (b) as resistant, yielding a binary outcome of susceptible vs. non-susceptible (including intermediate and resistant). Analysis accounted for year and injectable metaphylaxis administered and adjusted for clustering by pen (*N* = 16 pens). Statistically significant associations (*p* < 0.05) are bolded in the text. * Unconditional GEE accounting for clustering by pen. ** Median unbiased estimate, exact logistic regression.

**Table 4:** Summary of statistical analyses examining associations between metagenomic long-read sequencing data and subsequent BRD outcomes of interest for fall-placed beef calves in a research feedlot with eight pens of 100 calves repeated over two years. Models account for year and metaphylaxis and adjust for clustering by pen (*N* = 16 pens).

Calves with sequencing data included in model:	Associations of interest:	
All calves, 1 DOF (*n* = 840 calves)	Sequencing detection of bacteria at 1 DOF and association with BRD ≤13 DOF	
All calves, 13 DOF (*n* = 819 calves)	Sequencing detection of bacteria at 13 DOF and association with BRD 14–45 DOF	
BRD calves treated ≤ 13 DOF (*n* = 64 calves)	Sequencing detection of ARGs at 1 DOF and association with antimicrobial susceptibility at BRD treatment	
BRD calves treated from 14 to 45 DOF (*n* = 64 calves)	Sequencing detection of ARGs at 13 DOF and association with antimicrobial susceptibility at BRD treatment	
Outcome of interest:	Risk factors evaluated:	
Risk of BRD within first 13 DOF		Sequencing detection of individual bacteria (*M. haemolytica*, *P. multocida*, *H. somni*, *B. trehalosi*) at 1 DOF
Risk of BRD between 14 and 45 DOF		Sequencing detection of individual bacteria (*M. haemolytica*, *P. multocida*, *H. somni*, *B. trehalosi*) at 13 DOF
Antimicrobial susceptibility results at BRD treatment for any target organism (*M. haemolytica*, *P. multocida*, or *H. somni*):		Sequencing detection of at least one *Pasteurellaceae* (*M. haemolytica*, *P. multocida*, *H. somni*, or *B. trehalosi*) carrying specific ARGs:
Any macrolide resistance (tulathromycin, gamithromycin, tildipirosin, tilmicosin)	Analysis repeated for calves treated for BRD: ≤13 DOF 14 to 45 DOF for all comparisons	Analysis repeated for *mphE-msrE* *EstT*
15-membered ring macrolide resistance (tulathromycin or gamithromycin)		
16-membered ring macrolide resistance (tildipirosin or tilmicosin)		
Tetracycline resistance (oxytetracycline)		*tet(H)*

Antimicrobial susceptibility results were available for *M. haemolytica*, *P. multocida*, and *H. somni*, but not *B. trehalosi.*

## Data Availability

The genomic data presented in the study were published in the Sequence Read Archive as part of the companion paper [30]. Custom scripts can be accessed at https://github.com/coadunate/ASSETS_2, accessed on 30 September 2025.

## Supplementary Materials

The following supporting information can be downloaded at https://www.mdpi.com/article/10.3390/antibiotics14111098/s1, Tables S1. Summary of long-read metagenomic sequence statistics for *M. haemolytica*, *P. multocida*, *H. somni* and *B. trehalosi* from samples collected at arrival, 13 days on feed, and time of treatment for bovine respiratory disease; Table S2. Extended summary of frequency of samples with the antimicrobial resistance genes *msrE-mphE*, *EstT* or *tet(H)* detected by long-read metagenomic sequencing on at least one read identified as *M. haemolytica*, *P. multocida*, *H. somni* or *B. trehalosi* at arrival processing and 13 days on feed based on the dataset with matching AST data originally described for Bayesian latent class models in Abi Younes et al., 2025 [30]; Table S3. Summary of frequency of samples with the antimicrobial resistance genes *msrE-mphE*, *EstT* or *tet(H)* detected by long-read metagenomic sequencing on at least one read identified as *M. haemolytica*, *P. multocida*, *H. somni* or *B. trehalosi* at the time of first treatment for bovine respiratory disease (BRD).

## Abbreviations

The following abbreviations are used in this manuscript:

AMR	Antimicrobial Resistance
AMU	Antimicrobial Use
ARGs	Antimicrobial Resistance Genes
AST	Antimicrobial Susceptibility Testing
BHI	Brain Heart Infusion
BRD	Bovine Respiratory Disease
BW	Body Weight
CARD	Comprehensive Antimicrobial Resistance Database
CCAC	Canadian Council of Animal Care
CLSI	Clinical and Laboratory Standards Institute
DNPS	Deep Nasopharyngeal Swab
DOF	Days On Feed
GEE	Generalized Estimating Equations
MALDI-TOF-MS	Matrix-assisted Laser Desorption/Ionization Time-of-flight Mass Spectrometry
MIC	Minimal Inhibitory Concentration
PBS	Phosphate-Buffered Saline
ROC	Receiver Operating Characteristic Curve
