# Supplementary material for: Respiratory Bacteria and Antimicrobial Resistance Genes Detected by Long-Read Metagenomic Sequencing Following Feedlot Arrival, Subsequent Treatment Risk and Phenotypic Resistance in Feedlot Calves
Source: Antibiotics (Basel). 2025 Nov 1;14(11):1098. doi: 10.3390/antibiotics14111098 (PMC12649505; doi:10.3390/antibiotics14111098)
Supplement: Supplementary file 1 [file antibiotics-14-01098-s001.zip › antibiotics-3934787-supplementary.pdf]

Supplementary Materials

Respiratory Bacteria and Antimicrobial Resistance Genes Detected by Long-Read Metagenomic Sequencing Following Feedlot Arrival, Subsequent Treatment Risk and Phenotypic Resistance in Feedlot Calves

Jennifer N. Abi Younes[1], Lianne McLeod[1], Stacey R. Lacoste[1], Zhijian Chai[1], Emily K. Herman[2], E. Luke McCarthy[3], John R. Campbell[1], Sheryl P. Gow[4], Paul Stothard[5], Matthew G. Links[3], Simon J.G. Otto[6,7] and Cheryl L. Waldner[1*]

[1] Department of Large Animal Clinical Sciences, Western College of Veterinary Medicine, University of Saskatchewan, Saskatoon, SK S7N 5B4, Canada

[2] Department of Biochemistry and Medical Genetics, Max Rady College of Medicine, University of Manitoba, Winnipeg, MB R3E 0J9, Canada

[3] Department of Animal and Poultry Science, College of Agriculture and Bioresources, University of Saskatchewan, Saskatoon, SK S7N 5A8, Canada

[4] Canadian Integrated Program for Antimicrobial Resistance Surveillance, Public Health Agency of Canada, Saskatoon, SK S7N 5B4, Canada

[5] Department of Agricultural, Food, and Nutritional Science, Faculty of Agricultural, Life, and Environmental Sciences, University of Alberta, Edmonton, AB T6G 2P5, Canada

[6] HEAT-AMR (Human-Environment-Animal Transdisciplinary AMR) Research Group, School of Public Health, University of Alberta, Edmonton, AB T6G 2J7, Canada

[7] Centre for Healthy Communities, School of Public Health, University of Alberta, Edmonton, AB T6G 1C9, Canada

[*] Correspondence: cheryl.waldner@usask.ca

Supplementary Table S1. Summary of long-read metagenomic sequence statistics for *M. haemolytica*, *P. multocida*, *H. somni* and *B. trehalosi* from samples collected at arrival, 13 days on feed, and time of treatment for bovine respiratory disease.

Year	Bacteria	Median total base pairs (bp)	Median of per sample median read lengths (bp)	Median theoretical coverage (× genome size)[1]	Median number of reads	Percent of samples with ≥ 1 read
**2020**	*M. haemolytica*	4,358,976	1,342	1.6	1,907	100% (854)
(*n*=854	*P. multocida*	1,156,811	1,399	0.50	525	99.6% (851)
samples)	*H. somni*	10,571	1,127	0.004	7	88.4% (755)
	B. trehalosi	51,105	1,300	0.02	26	91.5% (781)
**2021**	*M. haemolytica*	4,426,431	3,692	1.6	828	100% (929)
(*n*=929	*P. multocida*	219,800	3,278	0.10	46	99.1% (921)
samples)	*H. somni*	8,458	2,125	0.004	2	75.2% (699)
	B. trehalosi	6,234	1,811	0.003	2	72.8% (676)

[1] Theoretical coverage for each sample was calculated by dividing the total read length for each of the bacteria of interest by the size of its reference genome (*M. haemolytica*: 2.8 Mb [NCBI GCF_002285575.1], *P. multocida*: 2.3 Mb [NCBI CF_002073255.2], *H. somni*: 2.3 Mb [NCBI GCF_000019405.1], *B. trehalosi*: 2.3Mb [NCBI GCF_000521725.1]).

Supplementary Table S2. Extended summary of frequency of samples with the antimicrobial resistance genes *msrE-mphE*, *EstT* or *tet(H)* detected by long-read metagenomic sequencing on at least one read identified as *M. haemolytica, P. multocida, H. somni* or *B. trehalosi* at arrival processing and 13 days on feed based on the dataset with matching AST data originally described for Bayesian latent class models in [30].

		**2020** (*n*=830 samples)				**2021** (*n*=827 samples)							
		**Tulathromycin metaphylaxis**				**Oxytetracycline metaphylaxis** (*n*=429)				**Tulathromycin metaphylaxis** (*n*=398)			
	**DOF**	***n*[1]**	***msrE-mphE***	***EstT***	***tet(H)***	***n*[1]**	***mrsE-mphE***	***EstT***	***tet(H)***	***n*[1]**	***mrsE-mphE***	***EstT***	***tet(H)***
M. haemolytica	Arrival processing	426	10 (2.3%)	5 (1.2%)	52 (12%)	214	0	2 (0.9%)	5 (2.3%)	198	0	0	7 (3.5%)
	13	404	134 (33%)	33 (8.2%)	57 (14%)	215	0	8 (3.7%)	45 (21%)	200	1 (0.5%)	27 (14%)	36 (18%)
P. multocida	Arrival processing	426	2 (0.5%)	1 (0.2%)	22 (5.2%)	214	0	0	2 (0.9%)	198	0	0	5 (2.5%)
	13	404	9 (2.2%)	3 (0.7%)	15 (3.7%)	215	0	0	25 (12%)	200	0	0	8 (4.0%)
H. somni	Arrival processing	426	1 (0.2%)	6 (1.4%)	20 (4.7%)	214	0	0	0	198	0	0	0
	13	404	59 (15%)	30 (7.4%)	10 (2.5%)	215	0	4 (1.9%)	11 (5.1%)	200	0	15 (7.5%)	8 (4.0%)
B. trehalosi	Arrival processing	426	1 (0.2%)	0	18 (4.2%)	214	0	0	1 (0.5%)	198	0	0	1 (0.5%)
	13	404	73 (18%)	3 (0.7%)	14 (3.5%)	215	0	0	6 (2.8%)	200	0	0	3 (1.5%)
Any of: *M. haemolytica*, *P. multocida*, *H. somni,* or *B. trehalosi*	Arrival processing	426	14 (3.3%)	9 (2.1%)	58 (14%)	214	0	2 (0.9%)	5 (2.3%)	198	0	0	9 (4.5%)
	13	404	140 (35%)	39 (9.7%)	60 (15%)	215	0	8 (3.7%)	54 (25%)	200	1 (0.5%)	30 (15%)	39 (20%)

This table builds on the table (Table 3) originally reported in Abi Younes et al., 2025 [30]. The original table included only *M. haemolytica*, *P. multocida*, and *H. somni* from arrival, 13 DOF, and 36 DOF. The present version has been expanded to include results for *B. trehalosi*. DOF – days on feed. [1] The *n* refers to the number of samples tested for ARGs at each DOF time point for that group (2020, 2021-oxytetracycline, and 2021-tulathromycin).

Supplementary Table S3. Summary of frequency of samples with the antimicrobial resistance genes *msrE-mphE*, *EstT* or *tet(H)* detected by long-read metagenomic sequencing on at least one read identified as *M. haemolytica, P. multocida, H. somni* or *B. trehalosi* at the time of first treatment for bovine respiratory disease (BRD).

	**2020** (*n*=27)				**2021** (*n*=101)							
	**Tulathromycin metaphylaxis**				**Oxytetracycline metaphylaxis** (*n*=88)				**Tulathromycin metaphylaxis** (*n*=13)			
	n[1]	***msrE-mphE***	***EstT***	***tet(H)***	***n***[1]	***mrsE-mphE***	***EstT***	***tet(H)***	***n***[1]	***mrsE-mphE***	***EstT***	***tet(H)***
M. haemolytica	27	13 (48%)	5 (19%)	7 (26%)	88	0	1 (1.1%)	24 (27%)	13	1 (7.7%)	3 (23%)	4 (31%)
P. multocida	27	1 (3.7%)	0	3 (11%)	88	0	0	12 (14%)	13	0	0	0
H. somni	27	3 (11%)	5 (19%)	4 (15%)	88	0	0	9 (10%)	13	0	1 (7.7%)	2 (15%)
B. trehalosi	27	2 (7.4%)	0	1 (3.7%)	88	0	0	8 (9.1%)	13	0	0	0
Any of:<br>*M. haemolytica, P. multocida, H. somni,* or *B. trehalosi*	27	13 (48%)	5 (19%)	10 (37%)	88	0	1 (1.1%)	27 (31%)	13	1(7.7%)	3 (23%)	5 (38%)

[1] The *n* refers to the number of samples tested for ARGs for that group (2020, 2021-oxytetracycline, and 2021-tulathromycin).
